# Data on administration of cyclosporine, nicorandil, metoprolol on reperfusion related outcomes in ST-segment Elevation Myocardial Infarction treated with percutaneous coronary intervention

**DOI:** 10.1016/j.dib.2017.07.033

**Published:** 2017-07-18

**Authors:** Gianluca Campo, Rita Pavasini, Giampaolo Morciano, Michael A. Lincoff, Michael C. Gibson, Masafumi Kitakaze, Jacob Lonborg, Amrita Ahluwalia, Hideki Ishii, Michael Frenneaux, Michel Ovize, Marcello Galvani, Dan Atar, Borja Ibanez, Giampaolo Cerisano, Simone Biscaglia, Brandon J. Neil, Masanori Asakura, Thomas Engstrom, Daniel A. Jones, Dana Dawson, Roberto Ferrari, Paolo Pinton, Filippo Ottani

**Affiliations:** a Cardiology Unit, Azienda Ospedaliero-Universitaria di Ferrara, Cona, FE, Italy; b Department of Morphology, Surgery and Experimental Medicine. Section of Pathology, Oncology and Experimental Biology, University of Ferrara, Ferrara, Italy; c Cleveland Clinic Coordinating Center for Clinical Research (C5Research), Cleveland, OH, USA; d PERFUSE Study Group, Cardiovascular Division, Department of Medicine, Beth Israel Deaconess Medical Center, Harvard Medical School, Boston, MA, USA; e Cardiovascular Division of Medicine, National Cardiovascular Centre, Suita, Osaka, Japan; f Department of Cardiology, Rigshospitalet, Copenhagen, Denmark; g Centre of Clinical Pharmacology, Barts NIHR Cardiovascular Biomedical Research Unit, William Harvey Research Institute, Barts & The London Medical School, Queen Mary University, London, UK; h Department of Cardiology, Nagoya University Graduate School of Medicine, Nagoya, Japan; i Norwich Medical School, University of East Anglia, Norwich, UK; j Clinical Investigation Center of Lyon, Lyon, France; k Unità Operativa di Cardiologia, Ospedale GB Morgagni, Forlì, Italy; l Department of Cardiology B, Oslo University Hospital Ullevall, and Faculty of Medicine, University of Oslo, Oslo, Norway; m Centro Nacional de Investigaciones Cardiovasculares Carlos III (CNIC), Madrid, Spain and Instituto de Investigación-Fundación Jiménez Díaz Hospital, Madrid, Spain; n Division of Cardiology, University of Florence, Careggi Hospital, Florence, Italy; o School of Medicine and Dentistry, University of Aberdeen, Aberdeen, UK; p Maria Cecilia Hospital, GVM Care & Research, E.S.: Health Science Foundation, Cotignola, Italy

**Keywords:** Reperfusion injury, Myocardial infarction, PCI, Cyclosporin, Nicorandil, Follow-up

## Abstract

Mortality and morbidity in patients with ST elevation myocardial infarction (STEMI) treated with primary percutaneous coronary intervention (PCI) are still high [1]. A huge amount of the myocardial damage is related to the mitochondrial events happening during reperfusion [2]. Several drugs directly and indirectly targeting mitochondria have been administered at the time of the PCI and their effect on fatal (all-cause mortality, cardiovascular (CV) death) and non fatal (hospital readmission for heart failure (HF)) outcomes have been tested showing conflicting results [3], [4], [5], [6], [7], [8], [9], [10], [11], [12], [13], [14], [15], [16]. Data from 15 trials have been pooled with the aim to analyze the effect of drug administration versus placebo on outcome [17]. Subgroup analysis are here analyzed: considering only randomized clinical trial (RCT) on cyclosporine or nicorandil [3], [4], [5], [9], [10], [11], excluding a trial on metoprolol [12] and comparing trial with follow-up length <12 months versus those with longer follow-up [3], [4], [5], [6], [7], [8], [9], [10], [11], [12], [13], [14], [15], [16]. This article describes data related article titled “Clinical Benefit of Drugs Targeting Mitochondrial Function as an Adjunct to Reperfusion in ST-segment Elevation Myocardial Infarction: a Meta-Analysis of Randomized Clinical Trials” [17].

**Specifications Table**

**Table:** 

Subject area	*Clinical research; meta-analysis*
More specific subject area	*Medicine; Cardiology; Reperfusion injury*
Type of data	*Figure*
How data was acquired	*Meta-analysis*
Data format	*Analyzed*
Experimental factors	*Ciclosporin or nicorandil, exclusion of metoprolol and follow-up length for reperfusion in ST elevation myocardial elevation treated with primary coronary intervention.*
Experimental features	*15 studies focused on drugs targeting mitochondrial function vs. placebo in patients undergoing primary PCI for STEMI, of which 3 with cyclosporine, 2 with nicorandil, only one study with metoprolol were retrieved from MEDLINE, Cochrane Library, Google Scholar and Biomed Central*
Data source location	*Italy, USA, Israel, Japan, Denmark, UK, France, Norway, Spain.*
Data accessibility	*Data is with this article*

**Value of the data**

• The use of cyclosporine or nicorandil at the time of primary percutaneous coronary angioplasty (PCI) on fatal (all-cause mortality, cardiovascular (CV) death) and non fatal (hospital readmission for heart failure (HF)) outcomes, show the absence of any potential benefit.
• Excluding a trial on metoprolol [12], which has a complex mechanism of action, not targeting only mitochondrial function, the pooled analysis on fatal and non fatal outcomes of the 14 studies did not changed.
• The analysis on follow-up length shows effects on hospital readmission for HF for trials with longer follow-up.
• These additional analyses should be the basis to plan further randomized clinical trials (RCTs) on reperfusion injury in ST elevation myocardial infarction (STEMI) patients undergoing PCI, focusing attention on other molecular mitochondrial targets.
• New RCTs on reperfusion injury should have a longer follow-up analysis.

## 1 Data

**Fig. 1:**
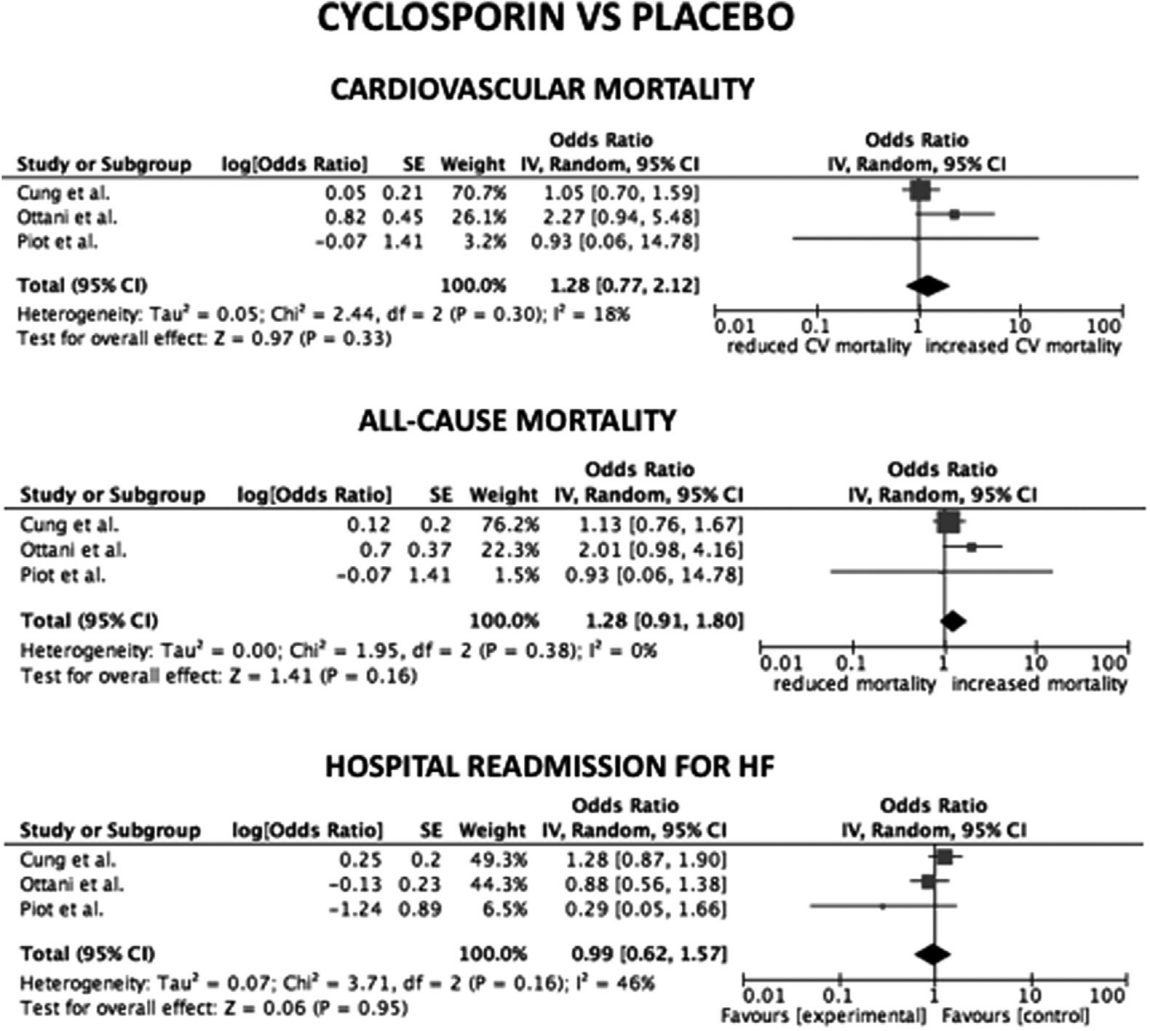
Forest plots on cardiovascular mortality, all-cause mortality and hospital readmission for HF in studies randomizing to cyclosporine vs. placebo. CV: cardiovascular.

**Fig. 2:**
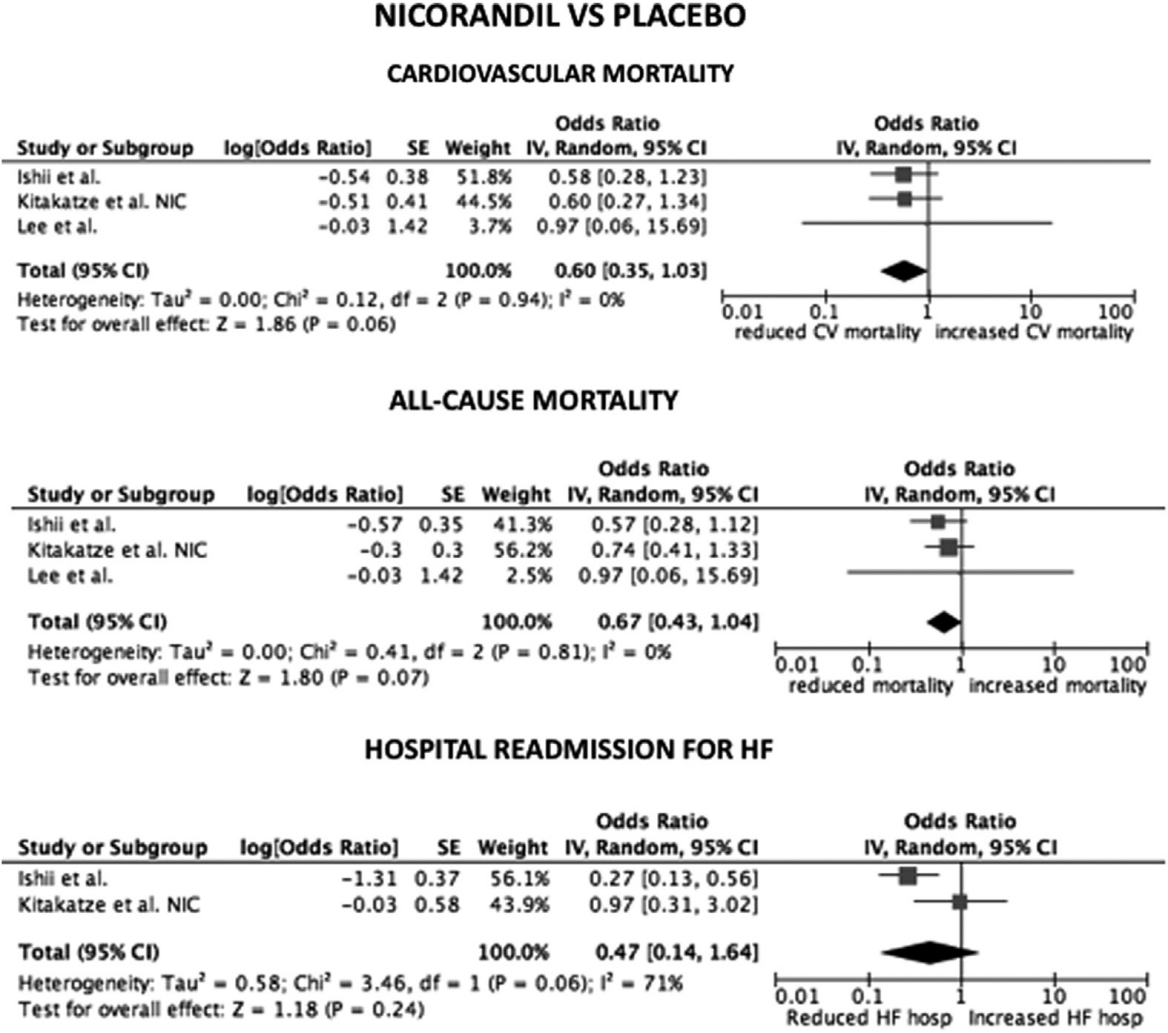
Forest plots on cardiovascular mortality, all-cause mortality and hospital readmission for HF in studies randomizing to nicorandil vs. placebo. CV: cardiovascular.

**Fig. 3:**
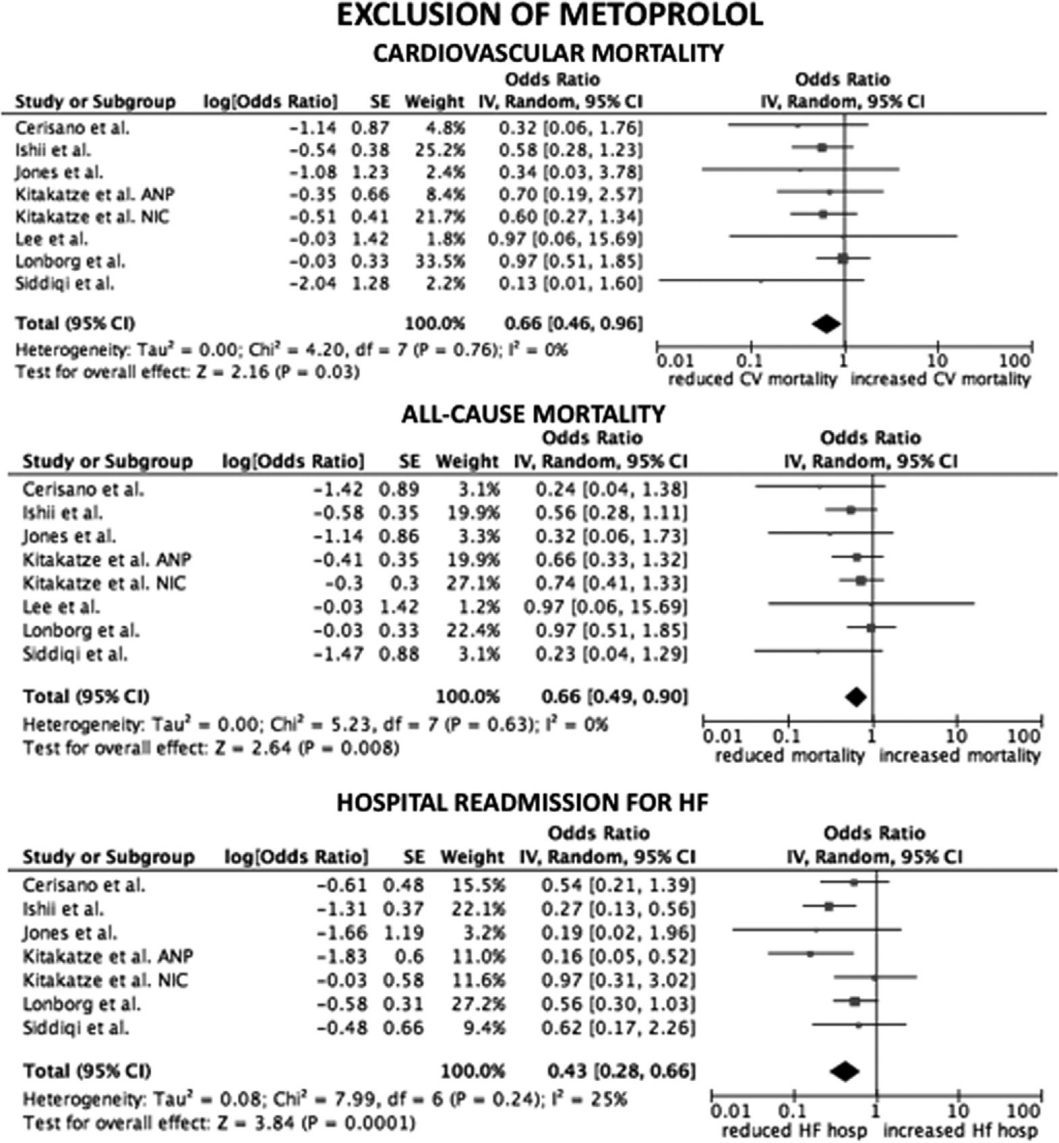
Forest plots on cardiovascular mortality, all-cause mortality and hospital readmission for HF in studies randomizing indirect/unspecific mechanism of action against mitochondrial component/pathway vs. placebo, excluding the study on metoprolol [12]. ANP: atrial natriuretic peptide. NIC: nicorandil. CV: cardiovascular. HF: heart failure. hosp: hospitalization.

**Fig. 4:**
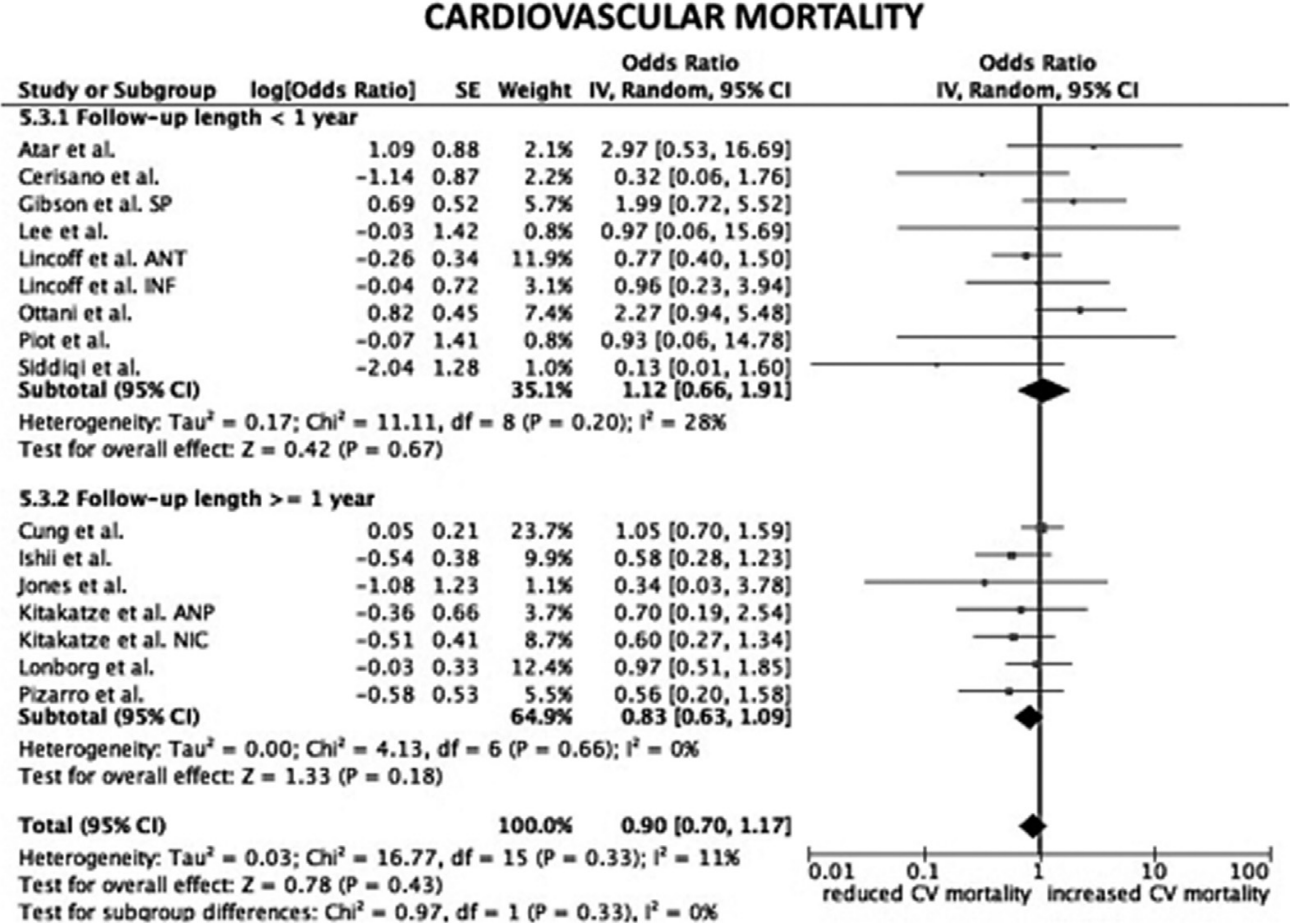
Forest plot on cardiovascular mortality after stratification of studies according to follow-up length. SP: safety population. ANT: anterior cohort. INF: inferior cohort. ANP: atrial natriuretic peptide. NIC: nicorandil. CV: cardiovascular.

**Fig. 5:**
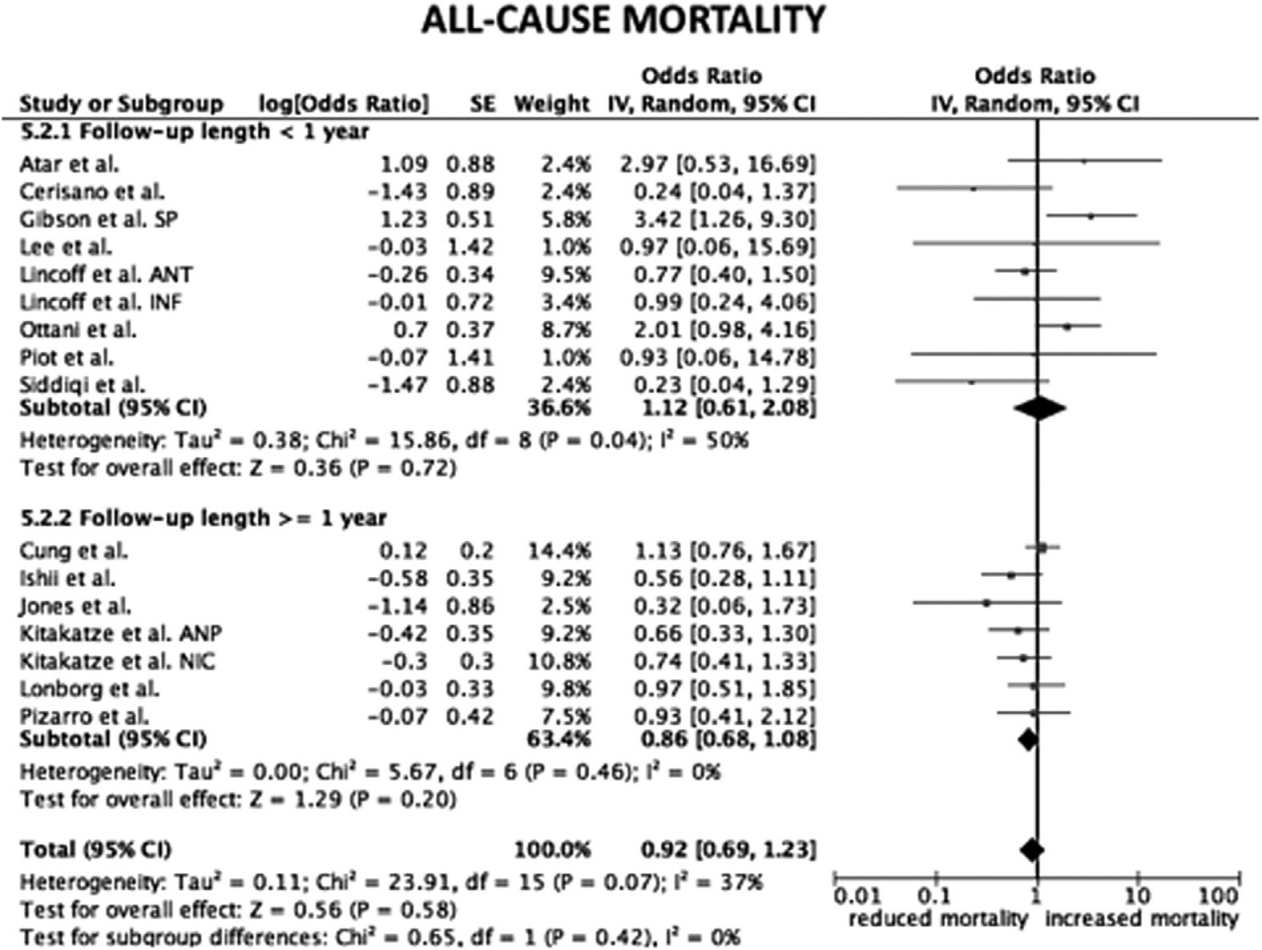
Forest plot on all-cause mortality after stratification of studies according follow-up length. SP: safety population. ANT: anterior cohort. INF: inferior cohort. ANP: atrial natriuretic peptide. NIC: nicorandil.

**Fig. 6:**
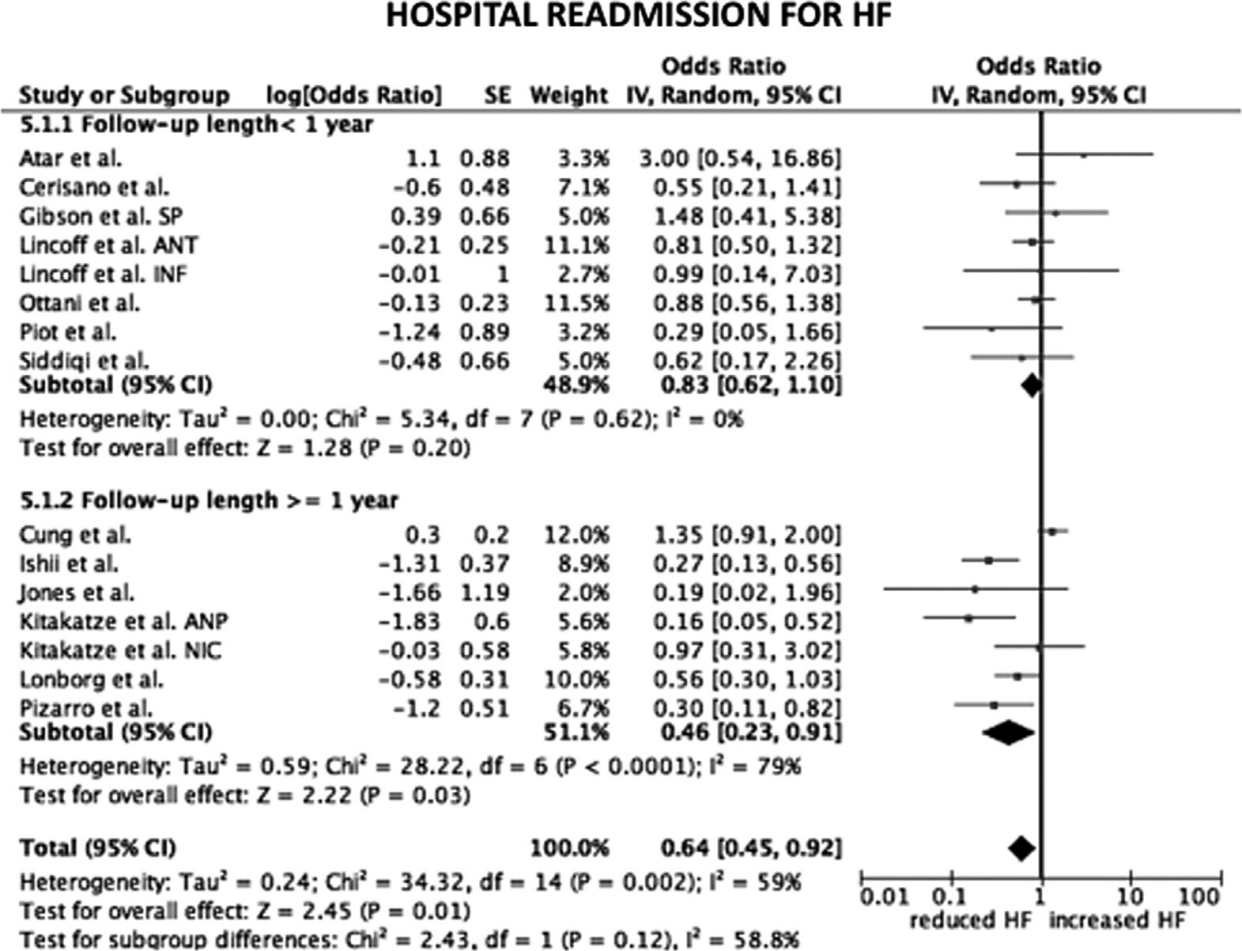
Forest plot on hospital readmission for heart failure after stratification of studies according follow-up length. SP: safety population. ANT: anterior cohort. INF: inferior cohort. ANP: atrial natriuretic peptide. NIC: nicorandil. HF: heart failure.

Considering only trial focused on cyclosporine versus placebo, the HR for CV mortality, all-cause mortality and hospital readmission for HF were not statistical significant (*p*=0.33; *p*=0.16; *p*=0.95, respectively) (Fig. 1). The same data are obtained considering only trials on nicorandil (*p*=0.06 for CV mortality; *p*=0.07 for all-cause death; *p*=0.2 for hospital readmission for HF) (Fig. 2). After the exclusion of the study on metoprolol from pooled analysis on trials with indirect/unspecific mechanism of action against mitochondrial component/pathway, the HR for CV death, all-cause death and hospital readmission for HF were significantly reduced (*p*=0.03; *p*=0.008; *p*=0.0001, respectively) (Fig. 3). Finally, the analysis on follow-up on all the studies included in the meta-analysis showed a reduction in hospital readmission for HF in studies with follow-up length ≥12 months (HR 0.46; 95% CI 0.45–0.92, *p*=0.03) (Fig. 4, Fig. 5, Fig. 6).

## 2 Experimental design, materials and methods

### 2.1 Search strategy

A systematic review and meta-analysis was performed following Preferred Reporting Items for Systematic reviews and Meta-Analyses (PRISMA) criteria [18], [19], [20], [21]. The protocol of this study was published on PROSPERO (CRD42016033085).

Papers were retrieved in MEDLINE, Cochrane Library, Google Scholar and Biomed Central. The terms searched were: (reperfusion injury) AND ((PCI) OR (percutaneous coronary intervention) OR (ST elevation myocardial infarction) OR (STEMI) OR (myocardial infarction)) [3], [4], [5], [6], [7], [8], [9], [10], [11], [12], [13], [14], [15], [16].

### 2.2 Selection criteria

Detailed description of selection criteria of the papers is described elsewhere [17]. In particular, we focused on i) RCTs ii) enrolling STEMI patients; with iii) reperfusion strategy by primary PCI; iv) comparison of agent/drug against RI vs. placebo/gold standard treatment.

### 2.3 Data abstraction, endpoints, contact with authors

We performed a pre-hoc stratification of studies according to mechanism of action targeting a mitochondrial component/pathway (direct/selective vs. indirect/unspecific) according to a recent overview [22]. The analyses were performed according to the following criteria: i) administration of cyclosporine, ii) administration of nicorandil, iii) follow-up length <12 vs. ≥12 months iv) indirect/unspecific drugs after exclusion of the study of Pizarro et al. [12]. The primary endpoint of the analysis was the incidence of cardiovascular death. Secondary endpoints were: all-cause death, hospital readmission for heart failure (HF).

### 2.4 Data analysis and synthesis

The endpoints were expressed as odds ratio (OR). Point estimates and standard errors were calculated and combined by the generic inverse variance method [23], computing risk estimates with 95% confidence intervals according to logarithmic transformation of the OR. A random effect model was used. Statistical heterogeneity was assessed with the Cochran's Q test and the I^2^ statistic [24]. To test the difference between sub-group analyses the Chi^2^ test has been used. Prometa (Internovi, Cesena, Italy) and RevMan 5 (The Cochrane Collaboration, The Nordic Cochrane Centre, Copenhagen, Denmark) software were used for statistical analyses.
