# Supplementary material for: Data on administration of cyclosporine, nicorandil, metoprolol on reperfusion related outcomes in ST-segment Elevation Myocardial Infarction treated with percutaneous coronary intervention
Source: Data Brief. 2017 Jul 18;14:197–205. doi: 10.1016/j.dib.2017.07.033 (PMC5537426; doi:10.1016/j.dib.2017.07.033)
Supplement: Supplementary file 1 — Supplementary material [file mmc1.pdf]

Conflicts of Interest Statement

Manuscript title: The role of cyclosporine, nicorandil, metoprolol and follow-up length on reperfusion related outcomes in ST elevation myocardial infarction treated with percutaneous coronary intervention: subgroup analyses.

The authors whose names are listed immediately below certify that they have NO affiliations with or involvement in any organization or entity with any financial interest (such as honoraria; educational grants; participation in speakers' bureaus; membership, employment, consultancies, stock ownership, or other equity interest; and expert testimony or patent-licensing arrangements), or non-financial interest (such as personal or professional relationships, affiliations, knowledge or beliefs) in the subject matter or materials discussed in this manuscript.

Author names:

Gianluca Campo, Rita Pavasini, Gianpaolo Morciano, Masafumi Kitakaze, Jacob Lonborg, Amrita Ahluwalia, Hideki Ishii, Michael Frenneaux, Michel Ovize, Marcello Galvani, Dan Atar, Borja Ibanez, Gianpaolo Cerisano, Simone Biscaglia, Brandon J Neil, Masanori Asakura, Thomas Engstrom, Daniel A Jones, Dana Dawson, Roberto Ferrari, Paolo Pinton, Filippo Ottani.

The authors whose names are listed immediately below report the following details of affiliation or involvement in an organization or entity with a financial or non-financial interest in the subject matter or materials discussed in this manuscript. Please specify the nature of the conflict on a separate sheet of paper if the space below is inadequate.

Author names:

- Lincoff receives research support from Kai Pharmaceuticals;
- Gibson receives research support from Stealth pharmaceuticals.

This statement is signed by all the authors to indicate agreement that the above information is true and correct (*a photocopy of this form may be used if there are more than 10 authors*):

Author's name (typed)	Author's signature	Date
GIANLUCA CAMPO	[illegible]	11/07/2017
RITA PAVASINI	[illegible]	10/07/2017
GIAMPAOLO MORCIANO	[illegible]	11/07/2017
MICHEAL LINCOFF	[illegible]	11/07/2017
MICHEAL GIBSON	[illegible]	11/07/2017
MASAFUMI KITAKAZE	[illegible]	10/07/2017
JACOB LONBORG	[illegible]	11/07/2017
AMRITA AHLUWALIA	[illegible]	11/07/2017
HIDEKI ISHII	[illegible]	10/07/2017
MICHEAL MERCEAUX	[illegible]	

This statement is signed by all the authors to indicate agreement that the above information is true and correct (*a photocopy of this form may be used if there are more than 10 authors*):

Author's name (typed)	Author's signature	Date
MICHAEL OVIZE		11/07/2017
MARCELLO GALVANI		10/07/2017
DAN ATAR		11/07/2017
BORJA IBANEZ		11/07/2017
GIAMPAOLO CERISANO		10/07/2017
SIMONE BISCAGLIA		11/07/2017
BRANDON J NEIL		11/07/2017
MASANORI NAKAMURA		11/07/2017
THOMAS ENGSTROM		10/07/2017
DANIEL A JONES		11/07/2017

This statement is signed by all the authors to indicate agreement that the above information is true and correct (*a photocopy of this form may be used if there are more than 10 authors*):

Author's name (typed)	Author's signature	Date
DANA DANSON		10/07/2017
ROBERTO FERRARI		11/07/2017
PAOLO PINTON		11/07/2017
FILIPPO OTTANI		10/07/2017
